# Cytobiological Alterations Induced by Celecoxib as an Anticancer Agent for Breast and Metastatic Breast Cancer

**DOI:** 10.34172/apb.2024.055

**Published:** 2024-06-29

**Authors:** Maher Monir. Akl, Amr Ahmed

**Affiliations:** ^1^Department of Chemistry, Faculty of Science, Mansoura University, 35516, Mansoura, Egypt.; ^2^The Public Health Department, Riyadh First Health Cluster, Ministry of Health, Saudi Arabia.

**Keywords:** Breast cancer subtypes, Celecoxib, COX-2 inhibition, Metastasis, Angiogenesis

## Abstract

Breast cancer remains a formidable public health challenge worldwide, characterized by its initiation within the breast’s diverse tissues, particularly the ducts and lobules. This malignancy is predominantly categorized into three subtypes based on receptor status and genetic markers: hormone receptor-positive, HER2-positive, and triple-negative. Each subtype exhibits distinct biological behaviors and responses to treatment, which significantly influence the prognosis and management strategies. The development and metastatic spread of breast cancer are complex processes mediated by interactions between tumor cells and the host microenvironment, involving various cellular and molecular mechanisms. This review highlights the potential therapeutic role of celecoxib, a selective cyclooxygenase-2 (COX-2) inhibitor, in addressing the multifaceted aspects of breast cancer progression. Specifically, celecoxib modulates angiogenesis by reducing the levels of vascular endothelial growth factor (VEGF) through decreased PGE2 production, enhances the immune response by alleviating PGE2-mediated immunosuppression, and inhibits metastasis by limiting the activity of matrix metalloproteinases (MMPs). These mechanisms collectively hinder tumor growth, immune evasion, and metastatic spread. By synthesizing recent findings and analyzing the impact of celecoxib on these pathways, this paper seeks to delineate the integrated approaches necessary for managing metastatic breast cancer effectively.

## Introduction

 Breast cancer, characterized by its site of origin within the diverse tissues of the breast, primarily the ducts and lobules, is a major global health concern due to its complex biological behavior and varied prognoses. This malignancy, which can metastasize or spread beyond the primary site, is categorized into several subtypes based on its receptor status and genetic features: hormone receptor-positive, HER2-positive, and triple-negative breast cancer (TNBC).^1^ Hormone receptor-positive breast cancer, which includes estrogen receptor (ER) and/or progesterone receptor (PR) positive subtypes, typically exhibits a slower progression and is more amenable to hormonal therapies such as tamoxifen or aromatase inhibitors. However, its capacity for late recurrences, even several years post initial diagnosis and treatment, underscores the necessity for long-term surveillance in clinical management.^2^ HER2-positive breast cancer, on the other hand, denotes the overexpression of the human epidermal growth factor receptor 2, a protein that promotes the growth of cancer cells. In the past, this subtype was associated with a poorer prognosis, but the advent of targeted therapies such as trastuzumab (Herceptin) has significantly improved outcomes. Nevertheless, the aggressive nature of HER2-positive tumors can lead to a more rapid progression and dissemination, often involving vital organs like the liver and lungs.^3^

 TNBC, defined by the absence of ER, PR, and HER2, is particularly notorious for its aggressive behavior and limited treatment options. The lack of targeted hormonal or HER2-directed therapies makes chemotherapy the primary treatment modality, albeit with a higher risk of recurrence and metastasis. TNBC is more likely to affect younger women and has a higher prevalence among African-American women. The metastatic pattern of TNBC often involves visceral organs and the central nervous system, complicating treatment and significantly worsening prognosis.^4^ The metastatic spread of breast cancer fundamentally involves the dissemination of tumor cells through lymphatic channels or the bloodstream to distant organs such as bones, liver, lungs, and brain. The process of metastasis is complex and influenced by the interaction between the tumor cells and the host microenvironment, facilitated by a cascade of molecular and cellular events including invasion, survival in the circulatory system, and colonization of new tissues.

 Each subtype of breast cancer thus presents unique challenges in terms of treatment, management, and research, necessitating a personalized approach to therapy that considers the biological characteristics of the tumor and the individual patient’s profile. Accurate diagnosis, timely and tailored treatment strategies, and ongoing research into the molecular underpinnings of breast cancer are critical to improving outcomes and survival rates for patients afflicted with this diverse and sometimes devastating disease.^5^

 In this context, the potential therapeutic role of celecoxib, a selective COX-2 inhibitor, emerges as a promising avenue. Celecoxib’s ability to modulate angiogenesis, immune response, and matrix metalloproteinase (MMP) activity presents significant implications for managing breast cancer’s progression and metastasis, particularly in addressing the challenges posed by each subtype. This review explores these mechanisms and evaluates the integrated therapeutic approaches necessary for effectively managing metastatic breast cancer.

## Methodology

 This review adopts a structured methodology to comprehensively explore the cytobiological alterations induced by celecoxib as an anticancer agent for breast and metastatic breast cancer. The methodology encompasses several key components aimed at elucidating the therapeutic potential of celecoxib and its underlying mechanisms of action.

 Firstly, a systematic literature search was conducted using reputable scientific databases such as PubMed, Scopus, and Web of Science. The search strategy employed a combination of relevant keywords and medical subject headings (MeSH) terms related to breast cancer, celecoxib, cytobiology, and anticancer mechanisms. Articles were screened based on predefined inclusion and exclusion criteria to ensure relevance and rigor in selecting pertinent studies.

 Secondly, selected articles were critically reviewed to extract pertinent data regarding celecoxib’s effects on cytobiological alterations associated with breast cancer. Data extraction included information on molecular pathways, cellular processes, and preclinical and clinical evidence supporting celecoxib’s anticancer properties.

 Thirdly, synthesized data were organized thematically to facilitate a coherent narrative that highlights the multifaceted impact of celecoxib on breast cancer biology. Emphasis was placed on elucidating the modulation of key cellular pathways involved in tumor progression, metastasis, and therapeutic resistance.

 Fourthly, gaps and inconsistencies in the existing literature were identified, providing insights into areas requiring further investigation and potential avenues for future research. This critical appraisal aimed to inform the development of evidence-based recommendations and hypotheses for advancing the understanding and clinical application of celecoxib in breast cancer management.

 Lastly, the findings were synthesized into a comprehensive narrative that integrates scientific evidence, theoretical frameworks, and clinical implications. This synthesis aims to provide clinicians, researchers, and policymakers with a nuanced understanding of celecoxib’s role as an adjunctive therapy in breast cancer treatment and its potential impact on patient outcomes.

## Breast cancer: development and metastasis mechanisms

 The development and metastasis of breast cancer involve a series of intricate cyto-biological mechanisms that transform a normal breast cell into a malignant one, characterized by uncontrolled growth, invasion, and eventually, dissemination to distant sites. Understanding these processes is crucial for developing targeted therapies and improving patient outcomes.

###  Genetic mutations and oncogenesis

**Figure 1:**
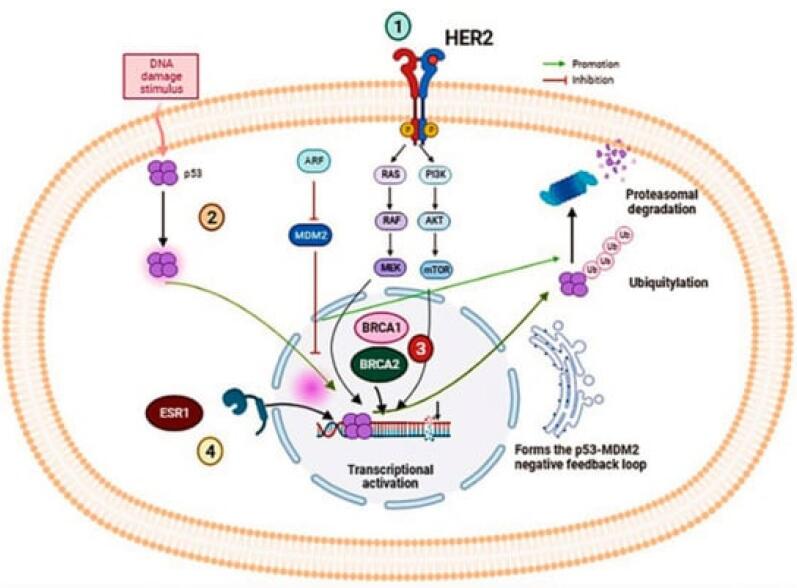
The illustration provides a detailed view of several critical signaling pathways involved in the oncogenesis of breast cancer, emphasizing the molecular complexity and potential therapeutic targets. The HER2/ERBB2 signaling pathway involves the receptor tyrosine kinase HER2, encoded by the ERBB2 gene, which is overexpressed in certain breast cancer subtypes and regulates cellular proliferation and survival. This pathway is a major target for treatments like monoclonal antibodies and tyrosine kinase inhibitors that disrupt aberrant HER2 signaling. Simultaneously, the p53 protein, crucial for controlling cell cycle and apoptosis, often mutates in breast cancer, leading to uncontrolled growth and resistance to cell death, thereby promoting tumor progression. Additionally, BRCA1 and BRCA2 genes play critical roles in DNA repair via homologous recombination, with mutations in these genes significantly increasing breast cancer risk due to impaired genomic stability. This genetic vulnerability has spurred targeted therapies and preventive strategies for those at high risk. Furthermore, Estrogen Receptor 1 (ESR1) functions as a transcription factor mediating estrogen's effects on cellular growth and differentiation, making estrogen receptor modulators and aromatase inhibitors foundational treatments in hormone receptor-positive breast cancers. Collectively, these pathways highlight the heterogeneity of breast cancer and the importance of personalized medicine in tailoring treatments to individual genetic and molecular profiles, underlining the intricate interplay of genetic regulation and molecular biology in cancer therapy.^7,8^ ReprintedUnder the terms and conditions of the Creative Commons Attribution (CC BY) license (https://creativecommons.org/licenses/by/4.0/)

 Cancer initiation often begins with genetic mutations in key regulatory genes, such as tumor suppressor genes (e.g., BRCA1, BRCA2, TP53) and oncogenes (e.g., HER2). These mutations can be inherited or acquired due to environmental factors, such as exposure to carcinogens or radiation. In breast cancer, mutations in BRCA1 and BRCA2 are particularly significant as they greatly increase the risk of developing the disease. BRCA1 and BRCA2 proteins play crucial roles in the homologous recombination (HR) repair pathway, a critical mechanism for repairing double-strand DNA breaks. Mutations in BRCA1 can disrupt its role in DNA damage detection, repair complex formation, and cell cycle checkpoint regulation, leading to defective DNA repair and genomic instability. Similarly, BRCA2 mutations impair its ability to facilitate the accurate repair of DNA double-strand breaks by mediating the loading of RAD51, resulting in the use of error-prone repair pathways. This genomic instability paves the way for additional mutations that drive tumorigenesis. TP53, another critical tumor suppressor gene, is commonly mutated in many cancers, including breast cancer. Mutations in TP53 lead to the production of a dysfunctional p53 protein that cannot effectively respond to DNA damage, allowing cells with damaged DNA to continue proliferating, thus accumulating further mutations and contributing to cancer progression. Furthermore, the oncogene HER2 is amplified or overexpressed in a subset of breast cancers, leading to excessive signaling through pathways such as PI3K/AKT and MAPK, which promote cell proliferation and survival. This oncogenic signaling exacerbates the aggressive behavior and poor prognosis often associated with HER2-positive breast cancers. Collectively, these genetic aberrations disrupt DNA repair and cell cycle regulatory mechanisms, driving the initiation and progression of breast cancer^6,7^ (Figure 1).

###  Cellular proliferation and tumor growth

**Figure 2:**
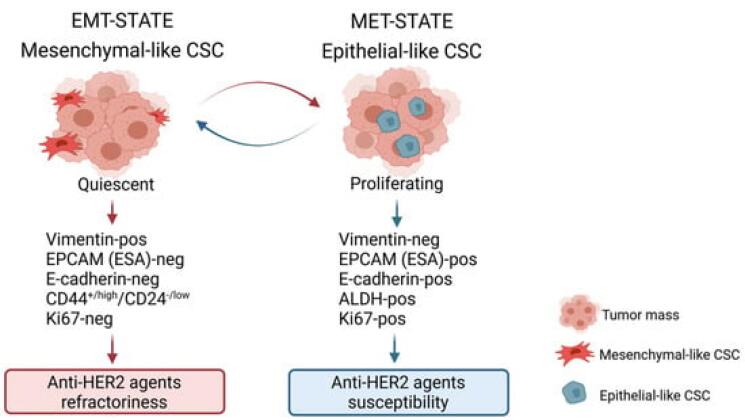
The characterization of breast cancer stem cell (BCSC) states reveals two distinct subtypes, each demonstrating unique phenotypic traits and responses to therapy, which underscore the complexity of targeting these cells in breast cancer treatment. The first type, mesenchymal BCSCs, are quiescent cells exhibiting a CD44 + /high/CD24−/low phenotype. These cells are particularly challenging to treat with anti-HER2 agents due to their inherent resistance. They are predominantly found at the periphery of the tumor mass, a state referred to as the EMT (epithelial-mesenchymal transition) state, which contributes to their ability to evade therapy and potentially enhance tumor invasion and metastasis. On the other hand, the epithelial type of BCSCs, characterized by high aldehyde dehydrogenase (ALDH) activity, demonstrates a proliferative nature and is more responsive to anti-HER2 therapies. These ALDH-positive cells are typically situated within the central regions of the tumor mass, where they contribute to the bulk of the tumor growth yet remain more accessible to therapeutic interventions. This dichotomy not only highlights the heterogeneity within breast cancer tumors but also emphasizes the need for tailored therapeutic approaches that consider the distinct biological behaviors and microenvironmental localizations of different BCSC types to effectively target and eradicate these cells^9^

 Once initiated, cancer cells gain the ability to proliferate uncontrollably due to alterations in signaling pathways that regulate cell growth and division. For instance, the overexpression of the HER2 protein in HER2-positive breast cancer leads to enhanced signaling through the PI3K/AKT and MAPK pathways, promoting rapid cell growth and division. Similarly, hormone receptor-positive breast cancers rely on hormonal signals (estrogen and progesterone) to drive their growth, exploiting normal cellular mechanisms for abnormal purposes^9^ (Figure 2).

###  Angiogenesis

**Figure 3:**
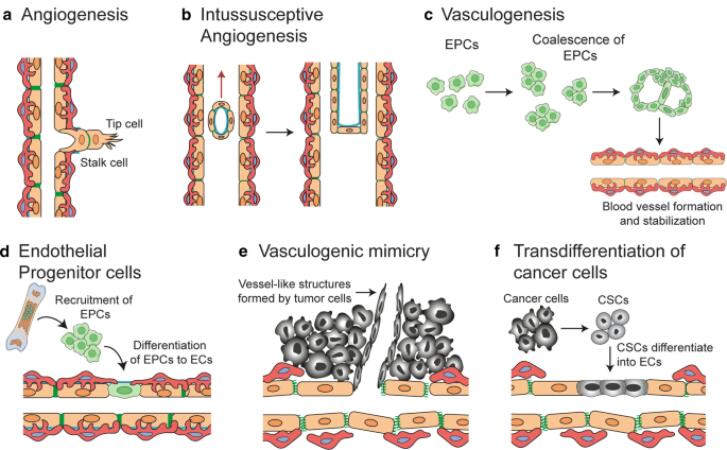
Mechanisms of blood vessel formation, critical both in normal tissues and tumors, involve several distinct processes that contribute to neo-vascularization. Sprouting angiogenesis; represents a fundamental mechanism where new sprouts form and extend from existing vessels, driven by tip cells, and eventually fuse to expand the vascular network. This form of angiogenesis is essential for the growth and healing of tissues. In contrast, intussusceptive angiogenesis; involves the splitting of pre-existing vessels into two separate channels, a process that rapidly increases vascular complexity without the need for new cell proliferation. Vasculogenesis, primarily occurring during prenatal development, entails the formation of blood vessels from endothelial progenitor cells that proliferate, form lumens, and assemble into new vasculature. In the context of tumors, additional specialized mechanisms come into play. Recruitment of endothelial progenitor cells; from the circulation allows tumors to form new blood vessels, supporting rapid tumor growth and metastasis. Vascular mimicry; occurs when tumor cells themselves form matrix-embedded, fluid-conducting networks, simulating the function of true blood vessels but without true endothelial lineage. Lastly, trans-differentiation of cancer stem cells (CSCs); into endothelial cells is a novel and significant mechanism whereby CSCs directly contribute to the tumor's vascular architecture. These diverse mechanisms underscore the complexity of blood vessel formation and highlight the adaptive strategies of both normal and tumor tissues in maintaining and expanding their vascular supply, which is crucial for delivering nutrients and oxygen and removing waste products^11^

 As the tumor grows, it requires nutrients and oxygen, which it obtains by promoting angiogenesis—the formation of new blood vessels. Tumor cells secrete angiogenic factors like vascular endothelial growth factor (VEGF), which stimulates nearby blood vessels to branch into the tumor, supplying it with the necessary resources to expand ^10^ (Figure 3).

###  Invasion and metastasis

 The progression from a localized tumor to a metastatic one hinges upon intricate biological mechanisms interwoven with cellular interactions and biochemical signaling pathways. This transition involves a series of orchestrated events, where proliferation instigates angiogenesis, thereby fostering invasion, and ultimately culminating in metastasis.

 Cancer cells, through the upregulation of enzymes like MMPs, undermine the integrity of cell-cell adhesion and the basement membrane, facilitating their detachment from the primary tumor site. This loss of adhesion enables cancer cells to infiltrate neighboring tissues by breaking down the extracellular matrix (ECM), a process pivotal for invasion.

 In breast cancer, the invasion-metastasis cascade unfolds through a sequence of meticulously regulated steps. Initially, cancer cells breach nearby lymphatic or blood vessels (intravasation), traversing the circulatory system. Subsequently, they extravasate from the bloodstream at distant metastatic sites, including the bones, liver, lungs, or brain. This metastatic dissemination relies heavily on dynamic interactions between cancer cells and the endothelial cells lining blood vessels, facilitating their transit and infiltration into new tissues.

**Figure 4:**
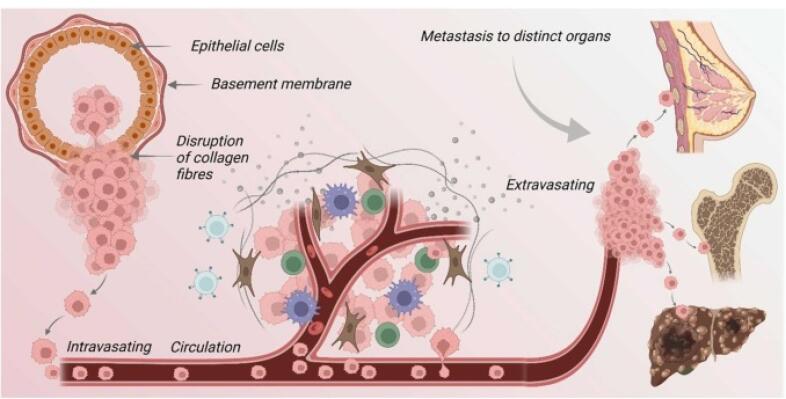
The pivotal role of basement membrane proteins in the migration and metastasis of cancer cells is increasingly recognized within the realm of oncological research. These proteins function as critical regulators in the transitional dynamics of cancer cells, from their primary site to distant organs. Initially, the transformation of normal epithelial cells leads to carcinoma in situ, characterized by a mere local invasion. As the disease progresses, there is a noticeable decrease in cellular adhesiveness coupled with an enhancement in migratory capabilities, enabling tumor cells to enter an invasive stage. Following this transition, the degradation of the basement membrane is a crucial step, as it facilitates the invasion of tumor cells into the surrounding stroma. Subsequently, these cells penetrate the circulatory system, traveling through blood vessels to colonize distant organs. This process includes the arrest of the viable cancer cells within the capillaries of target organs. Furthermore, an alternative pathway involves the direct entry of tumor cells into circulation, leading to the establishment of secondary tumors. This secondary tumor formation is supported by the proliferation of the tumor cells, induction of angiogenesis, and the modification of the local microenvironment to support the new tumor growth. Thus, the interaction between basement membrane proteins and cancer cells is fundamental to the understanding and potentially curbing the spread of cancer through metastasis^13^

 Upon reaching secondary sites, cancer cells encounter a foreign microenvironment, necessitating adaptive responses to survive and proliferate. This colonization process involves intricate signaling interactions and is often influenced by dysregulated immune responses, further shaping the metastatic niche^12^ (Figure 4).

###  Microenvironment and metastatic niche formation

 Cancer cells that succeed in forming metastases alter their new environment to support their survival and proliferation. This involves interacting with local cells to create a ‘metastatic niche’ that provides growth factors, protection from the immune response, and other supportive conditions.^14^

 The formation of a metastatic niche and the intricate role of the tumor microenvironment (TME) are pivotal in the metastasis of cancer, involving a complex interplay of cellular, molecular, and biochemical dynamics that govern the disease’s progression and dissemination. The TME comprises a heterogeneous array of cellular constituents, including neoplastic cells, fibroblasts, immune cells, and endothelial cells, alongside non-cellular elements such as cytokines, growth factors, and the ECM. This dynamic ensemble promotes tumor progression and metastatic capability via critical phenotypic transformations in tumor cells, notably the epithelial-to-mesenchymal transition (EMT). This process is essential as it enables epithelial cells to lose their polarity and adhesion, acquiring migratory and invasive properties fundamental to metastasis. On a molecular level, interactions between neoplastic cells and the ECM are crucial. MMPs, enzymes responsible for ECM degradation, facilitate neoplastic cell migration and invasion, while cytokines and growth factors such as TGF-β and VEGF drive angiogenesis, fostering the development of vascular networks that not only support tumoral expansion but also serve as conduits for metastatic cells. The establishment of metastatic niches at distant sites is orchestrated by complex preparatory changes led by disseminated tumor cells and primary tumor-secreted factors. These changes begin with the secretion of tumor-derived exosomes that modify the local ECM, making it conducive to metastatic colonization. Moreover, immune modulation occurs as tumor cells and bone marrow-derived cells manipulate the local immune response to create an immunosuppressive environment conducive to evasion from immune surveillance. This is accompanied by the recruitment and reprogramming of local stromal cells, such as fibroblasts and macrophages, to further support tumoral growth and survival. A nuanced understanding of these processes not only elucidates the underpinnings of metastasis but also highlights potential targets for therapeutic intervention. By disrupting key processes such as EMT, ECM degradation, angiogenesis, and immune modulation, novel therapeutic strategies could significantly reduce or even prevent the spread of cancer, marking a significant leap forward in oncological therapeutics.^15^

 Celecoxib, a well-known non-steroidal anti-inflammatory drug (NSAID), is primarily recognized for its selective inhibition of cyclooxygenase-2 (COX-2), an enzyme critical to the inflammatory process. While its anti-inflammatory properties are widely acknowledged, celecoxib has also garnered attention for its potential anti-cancer effects, particularly in the context of cancer pathogenesis and metastasis.^16^

## Mechanism of Action

###  COX-2 inhibition and apoptosis induction

**Figure 5:**
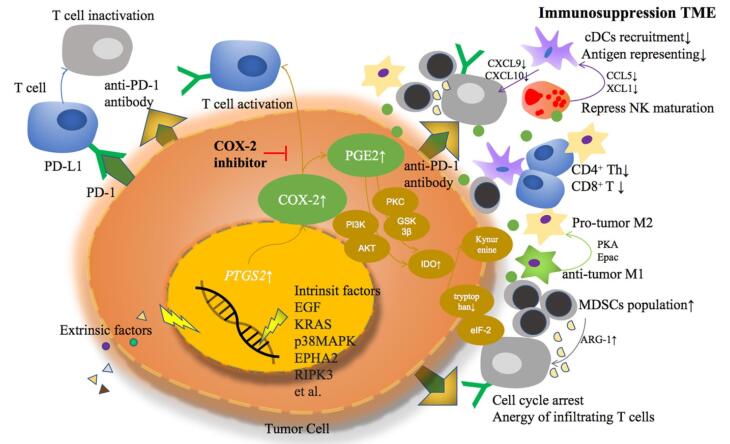
we observe the critical molecular pathways within tumor cells where both extrinsic and intrinsic factors catalyze the overexpression of Cyclooxygenase-2 (COX-2) and the subsequent overproduction of Prostaglandin E2 (PGE2). These molecular events are illustrated as a series of cascading steps leading from the cellular membrane to the nucleus, highlighting the activation of these pathways. The image effectively showcases how this overactivity contributes to the formation of an immunosuppressive tumor microenvironment, depicted by various signaling interactions among different cell types in the surrounding area. This diagram emphasizes the multifaceted impact of COX-2 and PGE2, linking them to the broader cellular processes that aid in immune evasion by the tumor^18^

 Celecoxib’s anti-cancer effects begin with the selective inhibition of COX-2. This enzyme, typically inducible in various cancers, including breast cancer, leads to increased production of prostaglandins, especially prostaglandin E2 (PGE2), which promotes angiogenesis, tumor growth, and metastasis. By targeting COX-2, celecoxib reduces PGE2 levels, thereby hindering these tumorigenic processes. Additionally, celecoxib can induce apoptosis in cancer cells by modulating apoptotic pathways, including the activation of caspases and the down-regulation of anti-apoptotic proteins like Bcl-2^17^ (Figure 5).

###  Angiogenesis inhibition

**Figure 6:**
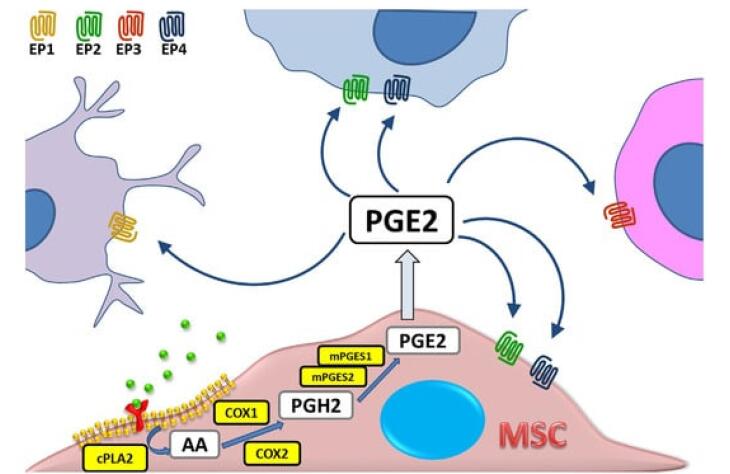
illustrates the biochemical pathway of prostaglandin E2 (PGE2) synthesis within a mesenchymal stromal cell (MSC). The pathway begins with arachidonic acid (AA), which is liberated from the cell membrane phospholipids by the action of cytosolic phospholipase A2 (cPLA). The free AA is then converted into Prostaglandin H2 (PGH2) through the enzymatic activity of cyclooxygenase (COX), also known by its other aliases, Prostaglandin endoperoxide synthase (PTGS) and prostaglandin G/H synthase (PGHS). Subsequently, PGH2 is transformed into PGE2 by the action of microsomal prostaglandin E synthases (mPGES). Once synthesized, PGE2 can exert its biological effects through interaction with one of its four specific receptors, EP1 through EP4, each mediating different signaling pathways and cellular responses. This schematic representation highlights the crucial steps involved in the biosynthesis of PGE2, emphasizing the enzymatic conversions and the specific enzymes involved in each step^20^

 Celecoxib, a specific COX-2 inhibitor, plays a pivotal role in the modulation of tumor biology by reducing the levels of PGE2, a key mediator in the TME. The decrease in PGE2 levels instigated by celecoxib directly impacts angiogenesis, the process by which new blood vessels form from pre-existing vessels, which is crucial for tumor growth. This effect is primarily mediated through a reduction in the production of VEGF, an essential pro-angiogenic factor. VEGF is instrumental in promoting the proliferation and migration of endothelial cells, which are central to angiogenesis. By lowering VEGF levels, celecoxib effectively impairs the tumor’s ability to develop and maintain its necessary blood supply. This compromised vascular support restricts the tumor’s access to vital nutrients and oxygen, ultimately inhibiting its growth and survival. Thus, the action of celecoxib not only targets inflammation but also significantly disrupts the angiogenic pathways, delivering a dual therapeutic strike against tumor progression^19^ (Figure 6).

###  Immune modulation

 Celecoxib, a selective COX-2 inhibitor, has been shown to potentially bolster the body’s immune defense against cancer by mitigating PGE2-mediated immunosuppression. PGE2, a bioactive lipid mediator derived from arachidonic acid metabolism, plays a pivotal role in modulating immune responses, inflammation, and tumorigenesis within the TME. Elevated levels of PGE2 have been associated with tumor-promoting activities, including angiogenesis, immune evasion, and metastasis. Mechanistically, PGE2 exerts its effects through interaction with its cognate receptors (EP1-EP4), leading to the activation of downstream signaling cascades such as the cyclic AMP (cAMP) pathway and the phosphoinositide 3-kinase (PI3K)/AKT pathway. By promoting the expression of pro-angiogenic factors such as VEGF, PGE2 facilitates the formation of new blood vessels, thereby enhancing tumor growth and metastatic spread.

 Similarly, VEGF represents a key regulator of angiogenesis, exerting its effects through binding to its receptors (VEGFR1-3) on endothelial cells. Activation of the VEGF pathway stimulates endothelial cell proliferation, migration, and tube formation, culminating in the formation of new blood vessels to support tumor growth and dissemination. Importantly, the dysregulation of VEGF signaling has been implicated in tumor angiogenesis, progression, and resistance to anti-cancer therapies.^21^

###  Metastasis inhibition via MMP regulation

 The metastatic spread of cancer involves complex processes where cancer cells must detach from the primary tumor, invade through the basement membrane, and eventually establish secondary tumors. MMPs play a crucial role in this process by degrading the ECM, facilitating cancer cell invasion and migration. Celecoxib indirectly affects MMP expression by reducing the levels of PGE2, a promoter of MMP expression. By limiting MMP activity, celecoxib inhibits the physical remodeling of the ECM, crucial for cancer cell invasion and metastasis.^22^

 Despite the promising therapeutic potential of celecoxib in targeting these pathways, the clinical use of celecoxib, especially as an anti-cancer agent, must be carefully managed due to potential cardiovascular risks associated with COX-2 inhibition, such as increased risk of heart attack and stroke. Therefore, the therapeutic application of celecoxib in oncology requires careful patient selection, monitoring, and a balanced consideration of its benefits against possible adverse effects.^23^

**Figure 7:**
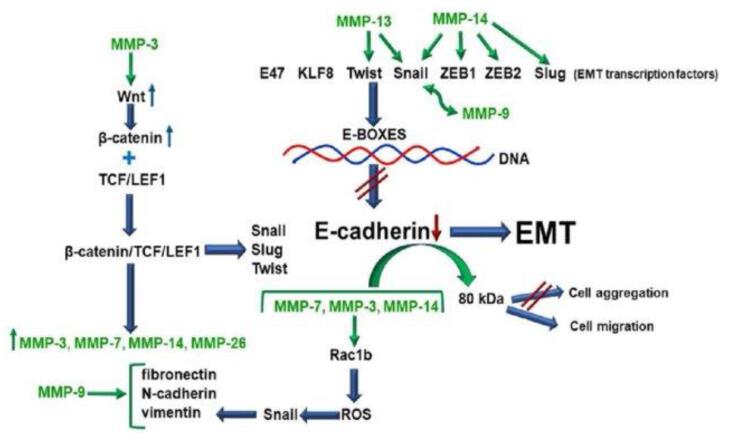
Matrix metalloproteinases (MMPs) play a crucial role in the process of epithelial-to-mesenchymal transition (EMT), a pivotal event in cancer progression and metastasis. The inhibition of E-cadherin synthesis and the disruption of its structural integrity are fundamental steps in initiating EMT. MMPs, including MMP-3, MMP-7, and MMP-14, contribute to this process by directly or indirectly affecting the regulation of EMT-associated transcription factors. These MMPs can act through pathways such as the Wnt/β-catenin signaling pathway or through the production of reactive oxygen species (ROS), leading to the upregulation of transcription factors that inhibit E-cadherin expression. Furthermore, specific MMPs disrupt E-cadherin on the cell surface, releasing fragments that functionally block E-cadherin in a paracrine manner, further impeding its tumor-suppressing effects. As a result of these processes, neoplastic cells lose their epithelial characteristics and acquire mesenchymal markers such as fibronectin, vimentin, N-cadherin, and the ability to synthesize collagen types I and III, along with several MMPs. This transformation not only facilitates the invasive and migratory capabilities of cancer cells but also significantly alters their interaction with the surrounding tumor microenvironment, promoting cancer progression and metastasis^25^

 In the realm of breast cancer therapy, the strategic targeting of metastatic processes remains a significant challenge. A recent meta-analysis exploring the efficacy of celecoxib, a selective COX-2 inhibitor, in mitigating metastatic breast cancer has synthesized findings from multiple studies to evaluate its therapeutic potential and safety profile. This analysis incorporated data from randomized controlled trials and observational studies that quantified the impact of celecoxib on metastasis rates, overall survival, and disease-free survival in breast cancer patients^24^ (Figure 7).

## Findings from the meta-analysis

 The pooled results indicated that celecoxib effectively reduces the expression of MMPs, enzymes critically involved in the degradation of the ECM, which facilitates tumor invasion and metastasis. By inhibiting MMP activity, celecoxib appears to impair the invasive capabilities of breast cancer cells, thereby potentially limiting the progression of metastatic disease. Furthermore, the analysis reported a significant association between celecoxib use and reduced angiogenesis, as evidenced by lowered levels of VEGF, which is crucial for the formation of new blood vessels needed for tumor growth and dissemination.^26^

## Discussion

 The findings from multiple studies suggest that celecoxib’s inhibition of COX-2 and subsequent effects on PGE2 levels offer a multifaceted approach to cancer therapy. By reducing angiogenesis through the downregulation of VEGF and modulating immune responses by decreasing PGE2-mediated immunosuppression, celecoxib enhances the body’s ability to combat tumor growth and spread. Moreover, the reduction in MMP expression facilitated by celecoxib may significantly retard the metastatic capabilities of breast cancer cells, addressing a critical challenge in the management of metastatic breast cancer. However, the clinical application of celecoxib in breast cancer treatment must consider the balance between its anti-cancer benefits and potential cardiovascular risks associated with long-term COX-2 inhibition. Future research should focus on optimizing dosing strategies, identifying patient cohorts that might benefit most from this therapy, and integrating celecoxib with other therapeutic modalities to enhance efficacy and safety.

## Conclusion

 Celecoxib presents a promising adjunct therapy in the management of breast cancer by targeting multiple pathways involved in tumor growth and metastasis. The ability of celecoxib to inhibit COX-2 and modulate both angiogenic and immune pathways offers a strategic advantage in treating particularly aggressive and metastatic forms of breast cancer. Nevertheless, the integration of celecoxib into clinical practice requires a thorough understanding of its molecular effects and a careful assessment of its risk-benefit ratio. As research progresses, the potential for celecoxib to serve as a component of a combined therapeutic regimen in breast cancer treatment is promising but necessitates further validation through clinical trials and comparative studies to establish its efficacy and safety comprehensively.

## Competing Interests

 The authors declare that there are no conflicts of interest.

## Ethical Approval

 Not applicable.
